# Prevalence of and risk factors for metabolic associated fatty liver disease in an urban population in China: a cross-sectional comparative study

**DOI:** 10.1186/s12876-021-01782-w

**Published:** 2021-05-10

**Authors:** Yu-ling Chen, Hao Li, Shu Li, Zhou Xu, Shen Tian, Juan Wu, Xin-yu Liang, Xin Li, Zi-li Liu, Jun Xiao, Jia-ying Wei, Chen-yu Ma, Kai-nan Wu, Liang Ran, Ling-quan Kong

**Affiliations:** 1 Department of Endocrine and Breast Surgery, The First Affiliated Hospital of Chongqing Medical University, Chongqing, 400016 China; 2 Department of Thyroid and Breast Surgery, Affiliated Hospital of North Sichuan Medical College, Nanchong, 637000 Sichuan China; 3 The Health Management Center of the First Affiliated Hospital of Chongqing Medical University, Chongqing, China

**Keywords:** Metabolic associated fatty liver disease, Nonalcoholic fatty liver disease, Metabolic syndrome, Ultrasonography

## Abstract

**Background:**

Metabolic associated fatty liver disease (MAFLD) is a new definition for liver disease associated with known metabolic dysfunction. Based on new diagnostic criteria, we aimed to investigate its prevalence and risk factors in Chinese population.

**Methods:**

We conducted this study in a health examination population who underwent abdominal ultrasonography in China. The diagnosis of MAFLD was based on the new diagnostic criteria. The characteristics of the MAFLD population, as well as the associations between MAFLD and metabolic abnormalities, were explored. Mann–Whitney U test and chi-square test were performed to compare different variables. Binary logistic regression was used to determine the risk factors for MAFLD.

**Results:**

Among 139,170 subjects, the prevalence of MAFLD was 26.1% (males: 35.4%; females: 14.1%). The prevalence based on female menopausal status, that is, premenopausal, perimenopausal, and postmenopausal, was 6.1%, 16.8%, and 30.2%, respectively. In different BMI groups (underweight, normal, overweight and obese), the prevalence was 0.1%, 4.0%, 27.4% and 59.8%, respectively. The proportions of abnormal metabolic features in the MAFLD group were significantly higher than those in the non-MAFLD group, as was the proportion of elevated alanine aminotransferase (ALT) (42.5% vs. 11%, *P* < 0.001). In nonobese individuals with MAFLD, the proportions of abnormal metabolic features were also all significantly higher than those in nonobese individuals without MAFLD. The prevalence of metabolic syndrome (MS), dyslipidaemia, and hyperuricaemia, respectively, in the MAFLD group (53.2%, 80.0%, and 45.0%) was significantly higher than that in the non-MAFLD group (10.1%, 41.7%, and 16.8%). Logistic regression revealed that age, BMI, waist circumference, ALT, triglycerides, fasting glucose, uric acid and platelet count were associated with MAFLD.

**Conclusions:**

MAFLD is prevalent in China and varies considerably among different age, sex, BMI, and female menopausal status groups. MAFLD is related to metabolic disorders, especially obesity, while metabolic disorders also play important roles in the occurrence of MAFLD in nonobese individuals. MAFLD patients exhibit a high prevalence of MS, dyslipidaemia, hyperuricaemia, and elevated liver enzymes. MAFLD tends to coexist with systemic metabolic disorders, and a deep inner relationship may exist between MAFLD and MS. Metabolic disorders should be considered to improve the management of MAFLD.

## Background

Metabolic associated fatty liver disease (MAFLD), formerly known as nonalcoholic fatty liver disease (NAFLD), is a new definition of liver disease associated with known metabolic dysfunction and is the most common chronic liver disease worldwide. NAFLD affects 24% of the adult population worldwide and poses a threat to human health [1]. NAFLD is generally considered to be closely related to obesity and multiple metabolic disorders, and can vary from hepatic steatosis to steatohepatitis, fibrosis or cirrhosis [2]. It is regarded as the hepatic manifestation of multisystem metabolic dysfunction [3]. Previously, the diagnosis of NAFLD was an exclusion diagnosis [3]; however, since research has progressed, NAFLD has been found to be derived from the potential state of multiple metabolic dysfunctions with complex pathophysiological characteristics, and its high prevalence in the general population makes it common to coexist with other liver diseases, which indicates that the exclusion criteria can no longer meet the current requirements for the diagnosis of the disease. Hence, in a recent international expert consensus, “MAFLD” was considered to be a better descriptor of liver disease associated with known metabolic dysfunction [4], and a set of positive diagnostic criteria were quickly released [5] so that MAFLD could be accurately, comprehensively and easily diagnosed. With the patient population being somewhat different from that of NAFLD, disease characteristics can be better manifested through the patient population diagnosed by new diagnostic criteria.

Therefore, this cross-sectional comparative study aims to investigate the prevalence and risk factors for MAFLD based on the new diagnostic criteria to better elucidate the association between MAFLD and multiple metabolic disorders, and provide a more accurate reference for the management and prevention of MAFLD.

## Methods

### Study population

This cross-sectional study used data from an urban population in Southwest China who participated in the health examination at the Quality Control Center of Health Examination in Chongqing, Southwest China, which is also known as the Health Management Center of the First Affiliated Hospital of Chongqing Medical University, from January 2015 to September 2018. In China, many organizations and companies may organize health check-ups for their employees and some individuals would also voluntarily go to medical institutions for regular health examinations to get known about their health condition. Therefore, the population of this study is a sample of an urban population in Southwest China. Our study included 139,170 participants, all of them had undergone comprehensive anthropometric measurements and clinical examinations, which included abdominal ultrasonography and the collection of fasting blood and urine samples. Repeat examinations of the same person were recognized by their unique health examination ID, and only one data set was randomly involved in the study. The exclusion criteria were incomplete data; age younger than 18 years; history of malignancy; history of oophorectomy or hysterectomy; and history of liver surgery or nephrectomy. The study was approved and supervised by The Ethics Committee of The First Affiliated Hospital of Chongqing Medical University (approval number: 2019-141) and was conducted in accordance with the Principles of the Helsinki Declaration. Requirement for informed consent was waived because all information was anonymous and retrospective.

### Anthropometric measurements and clinical examination

Blood pressure and anthropometric parameters, including height, weight and waist circumference, were measured using standardized procedures by trained examiners. Body mass index (BMI) was calculated as follows: BMI (kg/m^2^) = weight (kg)/height squared (m^2^). Venous blood samples of all participants were collected after at least 8 h of fasting and were analysed by standard laboratory procedures in the laboratory of The First Affiliated Hospital of Chongqing Medical University, which is certified by the College of American Pathologists (CAP No. 7215494). Abdominal ultrasound was performed using ultrasound scanners (Aplio500, Toshiba Medical Systems, Japan or HD11XE, Philips Medical Systems, USA). All abdominal ultrasonographies were performed and evaluated by experienced ultrasonographers at the Quality Control Center of Health Examination. Because the diagnosis of MAFLD does not involve the assessment of alcohol consumption and hepatitis virus, we did not include the two examinations in our study. Disease histories were checked in the health examination results of each participant. All data were recorded in the electronic medical record system of the Quality Control Center of Health Examination in Chongqing.

### Diagnosis of MAFLD

In our study, the diagnosis of MAFLD was based on the ultrasonically diagnosed hepatic steatosis and the presence of one of the following three criteria: overweight or obesity (defined as BMI ≥ 23 kg/m^2^ in Asians), type 2 diabetes mellitus, or metabolic dysregulation. Metabolic dysregulation was defined by the presence of at least two of the following metabolic risk abnormalities: 1) waist circumference ≥ 90/80 cm in Asian men and women; 2) blood pressure ≥ 130/85 mmHg or specific drug treatment; 3) plasma triglycerides ≥ 1.70 mmol/L or specific drug treatment; 4) plasma HDL-cholesterol < 1.0 mmol/L for men and < 1.3 mmol/L for women or specific drug treatment; 5) prediabetes (i.e., fasting glucose levels 5.6 to 6.9 mmol/L, or 2-h post-load glucose levels 7.8 to 11.0 mmol or HbA1c 5.7% to 6.4%; 6) plasma high-sensitivity C-reactive protein (hs-CRP) level > 2 mg/L; and 7) homeostasis model assessment (HOMA)-insulin resistance score ≥ 2.5 [5]. The diagnosis of hepatic steatosis on ultrasound was based on the presence of hepatorenal echo contrast, liver parenchymal brightness, deep attenuation, and vascular blurring [6, 7].

### Definitions

BMI groups of underweight (< 18.5 kg/m^2^), normal (≥ 18.5 kg/m^2^, < 23.0 kg/m^2^), overweight (≥ 23.0 kg/m^2^, < 25.0 kg/m^2^) and obese (≥ 25.0 kg/m^2^) were categorized based on the BMI criteria for Asians made by the WHO [8]. Metabolic syndrome (MS) was defined in accordance with the criteria by Joint Statement [9], which was based on the presence of at least 3 of the following components: (1) elevated waist circumference (≥ 90 cm for men and ≥ 80 cm for women); (2) elevated triglycerides (≥ 1.70 mmol/L) or drug treatment for elevated triglycerides; (3) reduced HDL-C (< 1.0 mmol/L for men and < 1.3 mmol/L for women) or drug treatment for reduced HDL-C; (4) elevated blood pressure (≥ 130/85 mm Hg) or drug treatment for hypertension; and (5) elevated fasting glucose (≥ 5.6 mmol/L) or drug treatment for elevated glucose. Dyslipidaemia was defined according to the guidelines for the prevention and treatment of dyslipidaemia in Chinese adults [10] as follows: a total cholesterol level of ≥ 5.2 mmol/L; LDL-C level of ≥ 3.4 mmol/L; HDL-C level of < 1 mmol/L; and triglycerides level of ≥ 1.7 mmol/L. Hyperuricaemia was defined as a uric acid level of ≥ 416 μmol/L for men or ≥ 357 μmol/L for women [11]. Menopausal status was defined as premenopausal period (≤ 45 years old), perimenopausal period (45–54 years old) and postmenopausal period (≥ 55 years old) according to the mean menopausal period for the Chinese female population [12, 13]. Elevated liver enzymes were defined as ALT > 35 IU/L and AST > 40 IU/L [14].

### Statistical analysis

All continuous variables were tested for normality and are described by medians (interquartile range) and proportions. The Mann–Whitney U test was performed to compare continuous variables due to their nonnormal distribution. For categorical variables, the chi-square test was performed to compare different variables. The specific prevalence of different age, BMI and female menopausal status groups and their 95% confidence intervals (CIs) were calculated. Binary logistic regression analysis was performed to explore the related risk factors for MAFLD. Odds ratios (ORs) and their 95% CIs were finally calculated. Binary logistic regression was performed using RStudio version 4.0.1, and other analyses were performed using SPSS 25. A two-tailed *p* value < 0.05 was considered statistically significant.

## Results

### General data for the participants

Of the 139,170 Chinese adults enrolled in the study, 78,176 subjects (56.2%) were males and 60,994 (43.8%) were females. The baseline characteristics of the study subjects are shown in Table 1. Compared with individuals without MAFLD, those with MAFLD were older, predominantly male and had higher values of body mass index (BMI), waist circumference, blood pressure, fasting glucose, total cholesterol, triglycerides, low-density lipoprotein cholesterol (LDL-C), albumin, total bilirubin, alanine aminotransferase (ALT), aspartate aminotransferase (AST), blood urea nitrogen (BUN), creatinine, uric acid, white blood cell count (WBC), red blood cell count (RBC), haemoglobin, platelet count, and hematocrit (HCT), and lower value of high-density lipoprotein cholesterol (HDL-C) (*P* < 0.05). Compared with males with MAFLD, females with MAFLD tended to be older and had higher values of systolic pressure, fasting glucose, total cholesterol, HDL-C, LDL-C, and platelet count (*P* < 0.05).

**Table 1:** Baseline characteristics of the study participants

Characteristics	Total (N = 139,170)			Male (N = 78,176)			Female (N = 60,994)			*P* value^†^
	MAFLD (N = 36,306)	Non-MAFLD (N = 102,864)	*P* value	MAFLD (N = 27,684)	Non-MAFLD (N = 50,492)	*P* value	MAFLD (N = 8622)	Non-MAFLD (N = 52,372)	*P* value	
Age (years)	47 (19)	42 (21)	< 0.001	45 (18)	43 (22)	< 0.001	54 (19)	41 (20)	< 0.001	< 0.001
Gender, male (%)	27,684 (76.3)	50,492 (49.1)	< 0.001	–	–	–	–	–	–	–
BMI (kg/m^2^)	26.18 (3.41)	22.27 (3.69)	< 0.001	26.35 (3.24)	23.05 (3.43)	< 0.001	25.59 (3.70)	21.48 (3.35)	< 0.001	< 0.001
Waist circumference (cm)	89 (9)	78 (12)	< 0.001	91 (9)	82 (10)	< 0.001	84 (9)	73 (9)	< 0.001	< 0.001
Systolic pressure (mmHg)	130 (24)	117 (22)	< 0.001	130 (22)	121 (21)	< 0.001	132 (28)	112 (21)	< 0.001	< 0.001
Diastolic pressure (mmHg)	81 (15)	72 (14)	< 0.001	82 (15)	75 (15)	< 0.001	78 (16)	69 (13)	< 0.001	< 0.001
Fasting glucose (mmol/L)	5.5 (1.0)	5.1 (0.7)	< 0.001	5.5 (1.0)	5.2 (0.6)	< 0.001	5.6 (1.0)	5.1 (0.6)	< 0.001	< 0.001
Total cholesterol (mmol/L)	5.02 (1.22)	4.64 (1.16)	< 0.001	4.98 (1.20)	4.68 (1.13)	< 0.001	5.13 (1.30)	4.61 (1.18)	< 0.001	< 0.001
Triglycerides (mmol/L)	2.04 (1.43)	1.10 (0.72)	< 0.001	2.12 (1.50)	1.26 (0.83)	< 0.001	1.79 (1.13)	0.97 (0.58)	< 0.001	< 0.001
HDL-C (mmol/L)	1.20 (0.36)	1.47 (0.45)	< 0.001	1.15 (0.32)	1.34 (0.39)	< 0.001	1.35 (0.39)	1.60 (0.43)	< 0.001	< 0.001
LDL-C (mmol/L)	3.30 (1.07)	2.83 (1.05)	< 0.001	3.29 (1.03)	2.97 (1.03)	< 0.001	3.32 (1.14)	2.70 (1.04)	< 0.001	0.001
Albumin (g/L)	47 (4)	47 (4)	< 0.001	48 (4)	47 (3)	< 0.001	46 (4)	46 (3)	< 0.001	< 0.001
Total bilirubin (μmol/L)	12.3 (5.9)	12.2 (5.8)	0.002	12.8 (6.1)	13.3 (6.3)	< 0.001	10.8 (4.7)	11.3 (5.0)	< 0.001	< 0.001
ALT (IU/L)	32 (24)	18 (13)	< 0.001	35 (26)	22 (15)	< 0.001	24 (16)	15 (8)	< 0.001	< 0.001
AST (IU/L)	24 (10)	20 (7)	< 0.001	25 (10)	22 (8)	< 0.001	22 (8)	19 (6)	< 0.001	< 0.001
BUN (mmol/L)	5.1 (1.6)	4.9 (1.7)	< 0.001	5.2 (1.6)	5.2 (1.7)	0.024	5.0 (1.7)	4.6 (1.6)	< 0.001	< 0.001
Creatinine (μmol/L)	73 (19)	66 (23)	< 0.001	77 (15)	78 (15)	< 0.001	56 (12)	56 (12)	0.059	< 0.001
Uric acid (μmol/L)	393 (121)	313 (115)	< 0.001	414 (110)	368 (96)	< 0.001	323 (89)	268 (73)	< 0.001	< 0.001
WBC (× 10^9^/L)	6.55 (1.97)	5.89 (1.85)	< 0.001	6.64 (1.96)	6.09 (1.88)	< 0.001	6.29 (1.94)	5.71 (1.79)	< 0.001	< 0.001
RBC (× 10^12^/L)	5.09 (0.62)	4.77 (0.70)	< 0.001	5.20 (0.49)	5.12 (0.50)	< 0.001	4.60 (0.44)	4.49 (0.43)	< 0.001	< 0.001
Haemoglobin (g/L)	155 (18)	144 (22)	< 0.001	159 (14)	157 (13)	< 0.001	138 (11)	134 (12)	< 0.001	< 0.001
Platelet count (× 10^9^/L)	211 (75)	210 (73)	< 0.001	207 (72)	200 (69)	< 0.001	226 (82)	219 (76)	< 0.001	< 0.001
HCT (%)	45.8 (4.8)	43.1 (6.0)	< 0.001	46.8 (3.6)	46.3 (3.7)	< 0.001	41.5 (3.3)	40.6 (3.3)	< 0.001	< 0.001
MS (%)	19,310 (53.2)	10,340 (10.1)	< 0.001	14,013 (50.6)	5675 (11.2)	< 0.001	5297 (61.4)	4665 (8.9)	< 0.001	< 0.001
Dyslipidaemia (%)	29,027 (80.0)	42,854 (41.7)	< 0.001	22,600 (81.6)	24,797 (49.1)	< 0.001	6427 (74.5)	18,057 (34.5)	< 0.001	< 0.001
Hyperuricaemia (%)	16,325 (45.0)	17,314 (16.8)	< 0.001	13,605 (49.1)	13,175 (26.1)	< 0.001	2720 (31.5)	4139 (7.9)	< 0.001	< 0.001

Data were described by medians (interquartile range) and proportions (%). *P* values were derived from Mann–Whitney U test or chi-square test

*MAFLD* metabolic associated fatty liver disease, *BMI* body mass index, *HDL-C* high-density lipoprotein cholesterol, *LDL-C* low-density lipoprotein cholesterol, *ALT* alanine aminotransferase, *AST* aspartate aminotransferase, *BUN* blood urea nitrogen, *WBC* white blood cell count, *RBC* red blood cell count, *HCT* hematocrit, *MS* metabolic syndrome

^†^The *P* value was calculated from the comparation between the groups of males with MAFLD (27,684) and females with MAFLD (8622)

### Prevalence of MAFLD and stratification by age, sex, menopausal status, and BMI

**Fig. 1:**
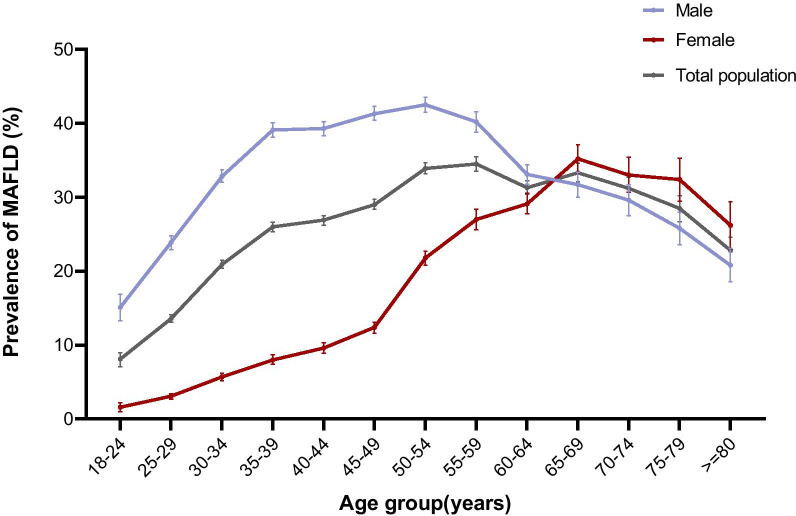
The age-specific prevalence of MAFLD. The age-specific prevalence of MAFLD and its 95% CIs in males, females and total population were calculated

**Fig. 2:**
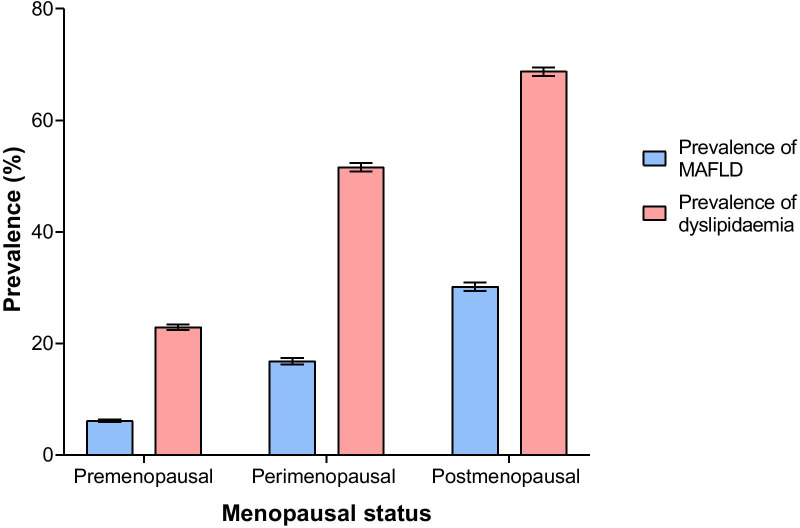
The prevalence of MAFLD and dyslipidaemia in females based on menopausal status. The prevalence of MAFLD and dyslipidaemia and its 95% CIs in females with different menopausal status were calculated

**Fig. 3:**
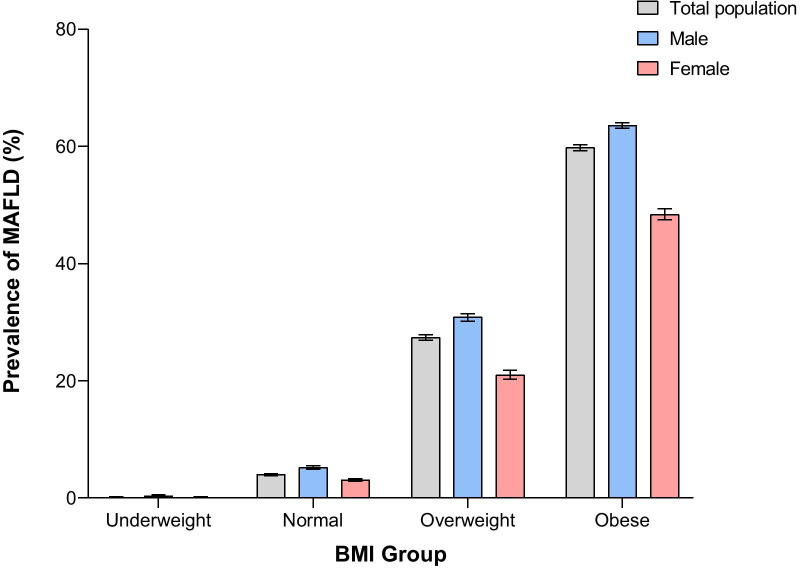
The prevalence of MAFLD based on BMI groups. The BMI-stratified MAFLD prevalence and its CIs in males, females and total population were calculated

Of the 139,170 participants, 36,306 (26.1%) were diagnosed with MAFLD, and a significant difference was found between males and females in the prevalence of MAFLD (35.4% vs. 14.1%, *P* < 0.001). After adjusting for age and sex, the overall prevalence of MAFLD was 23.8% (males: 32.3%, females: 13.4%). The age-specific prevalence of MAFLD is shown in Fig. 1. For the total population, the prevalence tended to rise with increasing age and then decrease, with a peak prevalence of 34.5% in the 55–59 age range. For females, the prevalence of MAFLD rose slowly in the 18–49 age range; however, it rose steeply after the age of 50, which was consistent with the appearance of the perimenopausal period and peaked at 35.2% in the 65–69 age range. For males, the prevalence rose rapidly between the ages of 18–39, rose slowly after 40 years and peaked at 42.5% in the 50–54 age range. The prevalence of MAFLD before the age of 65 was significantly higher in males than in females (36.2% vs. 12.2%, χ^2^ value = 9378.514, *P* < 0.001), whereas after the age of 65, the prevalence was significantly lower in males than in females (28.2% vs. 33.0%, χ^2^ value = 35.532, *P* < 0.001). In females, the prevalence of MAFLD was 6.1% [CI 5.9–6.4%] for the premenopausal group, 16.8% [CI 16.2–17.4%] for the perimenopausal group and 30.2% [CI 29.4–30.9%] for the postmenopausal group, and there was a significant difference in prevalence among the different groups (χ^2^ value = 4764.496, *P* < 0.001) (Fig. 2). In different BMI groups, the prevalence of MAFLD was 0.1% [CI 0.1–0.2%] for the underweight group, 4.0% [CI 3.8–4.1%] for the normal group, 27.4% [CI 26.9–27.9%] for the overweight group and 59.8% [CI 59.3–60.3%] for the obese group, and there was a significant difference among different BMI groups (χ^2^ value = 41,904.598, *P* < 0.001) (Fig. 3).

### Related risk factors for MAFLD and the association between MAFLD and metabolic disorders

**Fig. 4:**
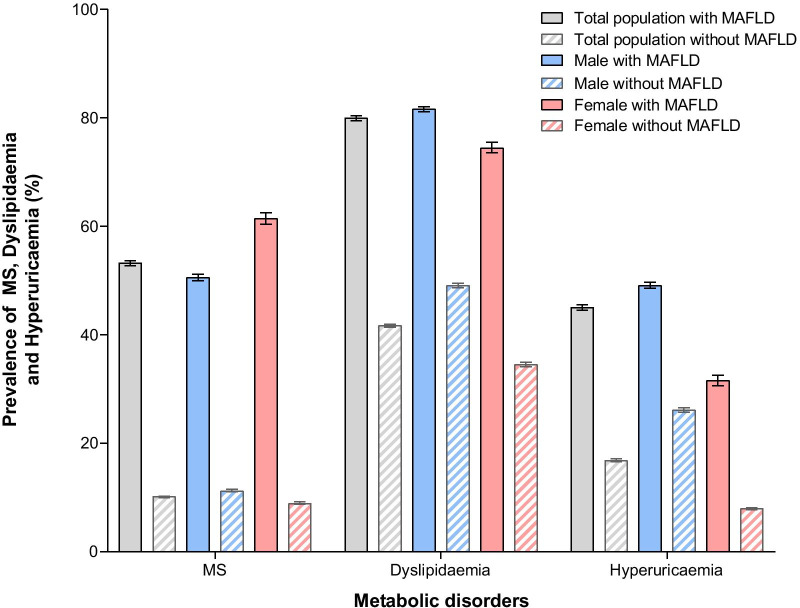
Prevalence of MS, dyslipidaemia and hyperuricaemia in individuals with and without MAFLD. The prevalence of MS, dyslipidaemia and hyperuricaemia and its CIs in individuals (total population, males and females) with and without MAFLD were calculated

The proportions of abnormal metabolic features in the MAFLD group were all significantly higher than those in the non-MAFLD group (*P* < 0.001, Table 2). The proportions of elevated liver enzymes, particularly elevated ALT, was also significantly higher in individuals with MAFLD than in individuals without MAFLD. Individuals with MAFLD were more likely to have multiple metabolic disorders, and the prevalence of MS, dyslipidaemia and hyperuricaemia in individuals with MAFLD was all significantly higher than that in individuals without MAFLD (Fig. 4) (for MS: 53.2% [CI 52.7–53.7%] vs. 10.1% [CI 9.9–10.2%], χ^2^ value = 29,779.866, *P* < 0.001; for dyslipidaemia: 80.0% [CI 79.5–80.4%] vs. 41.7% [CI 41.4–42.0%], χ^2^ value = 15,754.446, *P* < 0.001; for hyperuricaemia: 45.0% [CI 44.5–45.5%] vs. 16.8% [CI 16.6–17.1%], χ^2^ value = 11,587.748, *P* < 0.001). Notably, for males with MAFLD, the prevalence of dyslipidaemia and hyperuricaemia was significantly higher than that in females with MAFLD (for dyslipidaemia: 81.6% [CI 81.2–82.1%] vs. 74.5% [CI 73.6–75.5%], χ^2^ value = 206.391, *P* < 0.001; for hyperuricaemia: 49.1% [CI 48.6–49.7%] vs. 31.6% [CI 30.6–32.5%], χ^2^ value = 822.636, *P* < 0.001), whereas the prevalence of MS in males with MAFLD was significantly lower than that in females with MAFLD (50.6% [CI 50.0–51.2%] vs. 61.4% [CI 60.4–62.5%], χ^2^ value = 309.026, P < 0.001). Moreover, the prevalence of MAFLD increased with increasing numbers of MS components individuals had (Table 3). For individuals with and without MS, there was also a significant difference in the prevalence of MAFLD (65.1% [CI 64.6–65.7%] vs. 15.5% [CI 15.3–15.7%], χ^2^ value = 29,779.866, *P* < 0.001). In the binary logistic regression, our results revealed that eight variables were closely correlated with MAFLD, including age, BMI, waist circumference, ALT, triglycerides, fasting glucose, uric acid and platelet count (Table 4). Among these variables, triglycerides, BMI and fasting glucose had the most significant associations with MAFLD, exhibiting the highest OR values of 1.776, 1.476 and 1.403, respectively.

**Table 2:** Proportions of abnormal metabolic features and elevated liver enzymes among 139,170 participants

Characteristics	Total (N = 139,170)			Non-obese subjects (N = 97,079)			Obese with MAFLD (n [%]) (N = 25,163)	*P* value^†^
	With MAFLD (n [%]) (N = 36,306)	Without MAFLD (n [%]) (N = 102,864)	*P* value	Non-obese with MAFLD (n [%]) (N = 11,143)	Non-obese without MAFLD (n [%]) (N = 85,936)	*P* value		
Elevated waist circumference	23,038 (63.5)	18,840 (18.3)	< 0.001	3337 (29.9)	8238 (9.6)	< 0.001	19,701 (78.3)	< 0.001
Elevated systolic pressure	18,610 (51.3)	25,477 (24.8)	< 0.001	4999 (44.9)	18,163 (21.1)	< 0.001	13,611 (54.1)	< 0.001
Elevated diastolic pressure	13,487 (37.1)	14,824 (14.4)	< 0.001	3334 (29.9)	10,158 (11.8)	< 0.001	10,153 (40.3)	< 0.001
Elevated triglycerides	23,574 (64.9)	20,044 (19.5)	< 0.001	7182 (64.5)	14,143 (16.5)	< 0.001	16,392 (65.1)	0.204
Reduced HDL-C	10,042 (27.7)	12,336 (12.0)	< 0.001	3046 (27.3)	9301 (10.8)	< 0.001	6996 (27.8)	0.359
Elevated fasting glucose	17,165 (47.3)	21,259 (20.7)	< 0.001	5347 (48.0)	15,835 (18.4)	< 0.001	11,818 (47.0)	0.073
Elevated total cholesterol	15,287 (42.1)	28,121 (27.3)	< 0.001	4885 (43.8)	22,537 (26.2)	< 0.001	10,402 (41.3)	< 0.001
Elevated LDL-C (mmol/L)	16,386 (45.1)	25,543 (24.8)	< 0.001	5114 (45.9)	19,641 (22.9)	< 0.001	11,272 (44.8)	0.052
Elevated ALT	15,426 (42.5)	11,443 (11.1)	< 0.001	3816 (34.2)	8032 (9.3)	< 0.001	11,610 (46.1)	< 0.001
Elevated AST	3443 (9.5)	2593 (2.5)	< 0.001	774 (6.9)	1963 (2.3)	< 0.001	2669 (10.6)	< 0.001

Data were described by proportions (%). *P* values were derived from chi-square test

Elevated waist circumference: ≥ 90 cm for men and ≥ 80 cm for women. Elevated systolic pressure: ≥ 130 mmHg. Elevated diastolic pressure: ≥ 85 mmHg. Elevated triglycerides: ≥ 1.70 mmol/L. Reduced HDL-C: < 1.0 mmol/L for men and < 1.3 mmol/L for women. Elevated fasting glucose: ≥ 5.6 mmol/L. Elevated total cholesterol: ≥ 5.2 mmol/L. Elevated LDL-C: ≥ 3.4 mmol/L. Elevated ALT: > 35 IU/L. Elevated AST: > 40 IU/L

*MAFLD* metabolic associated fatty liver disease, *HDL-C* high-density lipoprotein cholesterol, *LDL-C* low-density lipoprotein cholesterol, *ALT* alanine aminotransferase, *AST* aspartate aminotransferase

^†^The *P* value was calculated from the comparation between the groups of Obese with MAFLD (25,163) and Non-obese with MAFLD (11,143)

**Table 3:** The prevalence of MAFLD in individuals with different number of MS components

Number of MS risk components	Total population	Individuals with MAFLD	Prevalence (%)
0	45,255	1471	3.3
1	36,339	5218	14.4
2	27,926	10,307	36.9
3	18,353	10,758	58.6
4	9033	6685	74.0
5	2264	1867	82.5

*MAFLD* metabolic associated fatty liver disease, *MS* metabolic syndrome

**Table 4:** Results of binary logistic regression of MAFLD and the tested variables

Variable	*P* value	OR	95% CI of OR
Age (years)	0.015	1.018	1.001–1.033
BMI (kg/m^2^)	0.000	1.476	1.320–1.660
Waist circumference (cm)	0.006	1.057	1.017–1.102
ALT (IU/L)	0.009	1.023	1.002–1.043
Triglycerides (mmol/L)	0.000	1.776	1.238–2.257
Fasting glucose (mmol/L)	0.000	1.403	1.116–1.839
Uric acid (μmol/L)	0.001	1.003	1.001–1.006
Platelet count (× 10^9^/L)	0.014	1.004	1.000–1.007

*OR* odds ratio, *CI* confidence interval, *MAFLD* metabolic associated fatty liver disease, *BMI* body mass index, *ALT* alanine aminotransferase

### Association between nonobese individuals and MAFLD

Among the nonobese population, the prevalence of MAFLD was 11.5% (males: 16.4%, females: 6.9%). The proportions of abnormal metabolic features and elevated liver enzymes in nonobese individuals with MAFLD were all significantly higher than those in nonobese individuals without MAFLD (*P* < 0.001) (Table 2). Compared with obese individuals with MAFLD, the proportions of patients with elevated waist circumference, elevated systolic and diastolic pressure and elevated liver enzymes were significantly lower in the nonobese MAFLD group, whereas no significant differences were found for elevated triglycerides, elevated LDL-C, reduced HDL-C and elevated fasting glucose between the two groups.

## Discussion

In the present study, the prevalence and risk factors for MAFLD were explored, and significant differences in the prevalence of MAFLD among groups based on sex, age, BMI and female menopausal status were revealed. To our knowledge, this study is the first to focus on the prevalence and associated metabolic characteristics of MAFLD in an urban Chinese population since the new definition of MAFLD was established [4, 5].

The age-specific prevalence shown in Fig. 1 revealed that males still predominated in the population with MAFLD. Moreover, for males, they were at an increased risk of MALFD at a younger age as evidenced by the rapid increase in the prevalence of MAFLD in the 18–39 age group, which should be given special attention. The peak prevalence in the 50–54 age group also indicated that MAFLD is more prevalent in their middle ages. We observed that older men had a lower prevalence of MAFLD than middle-aged men. Possible reasons for this result might include the following: some individuals may die of other diseases at older ages as fatty liver can significantly increase overall mortality [15], and thus these individuals are not counted as part of the MAFLD population; compared with older men who typically retire, middle-aged men who are at the peak of their careers may experience more pressure and engage in social behaviour that may lead to unhealthy lifestyles, which can increase their risk of having metabolic disorders. In females, the trend of prevalence differed from males. We observed that the prevalence of males rose rapidly during younger ages, rose slowly in their middle ages and then declined. While for females, the prevalence rose slowly during younger ages and then rose rapidly after the age of 45, which was consistent with the emergence of the perimenopausal period. Moreover, between the ages of 45 and 69, the prevalence in males showed a downward trend, whereas the prevalence in women still rose rapidly. These trends differences between sexes suggested that there might be a certain correlation between MAFLD and female menopausal status. Previous studies have found that a decrease in oestrogen in perimenopausal and postmenopausal women can lead to fat redistribution and thus cause metabolic disorders, including dyslipidaemia and glucose intolerance [16]. Our study also found that the prevalence of dyslipidaemia in females increased from the premenopausal period to the perimenopausal period and then to the postmenopausal period, which paralleled the rising prevalence of MAFLD in females in the three menopausal status groups (Fig. 2). This result indicates that the increase in MAFLD prevalence in women may be related to dyslipidaemia and metabolic disorders caused by a decline in oestrogen levels. Studies have also found that oestrogen might have favourable effects on lipid metabolism in the liver [17], which might be a protective factor against fatty liver in females [18]. Therefore, in combination with our findings and previous conclusions, oestrogen may also be a protective factor for females with MAFLD, and low oestrogen levels during the perimenopausal and postmenopausal periods may be an important risk factor for MAFLD in females.

Previous studies have found that the presence of NAFLD is closely correlated with components of MS, such as obesity, insulin resistance, hypertension and dyslipidaemia, and is considered to be the liver manifestation of MS [19]. Our study also found that after stratification by BMI, the prevalence of MAFLD increased sharply with increasing BMI, reaching 59.8% in obese individuals (Fig. 3). In the binary logistic regression analysis (Table 4), BMI and waist circumference were also significantly associated with MAFLD, indicating that obesity is closely associated with MAFLD and that obesity management should be emphasized, as weight loss has been proven to reduce steatosis [20].

In individuals with MAFLD, the proportions of abnormal metabolic features were all significantly higher than those in individuals without MAFLD (Table 2), confirming that MAFLD is closely associated with MS components, including abdominal obesity, hypertension, dyslipidaemia, and dysglycaemia. Among them, in addition to elevated waist circumference, the most significant difference was found in elevated triglycerides, and triglycerides were also shown to be significantly associated with MAFLD in the logistic regression (Table 4), with the highest OR value of 1.776, which suggests that elevated triglycerides may be an important risk factor for MAFLD. Moreover, the difference in the proportion of subjects with elevated fasting glucose was also highly significant, and fasting glucose was also significantly associated with MAFLD in the logistic regression with an OR value of 1.403, which is consistent with a previous study that showed a correlation between fatty liver and dyslipidaemia and dysglycaemia [21], indicating that elevated fasting glucose may also be an important risk factor for MAFLD. Previous studies have shown that NAFLD is not only closely correlated with cardiovascular and renal diseases associated with MS but also precedes the presentation of metabolic derangements [22], while a recent article found that compared with NAFLD, MAFLD can better identify patients with more metabolic disorders and a higher risk of disease progression [23]. In our study, we found a high prevalence of metabolic abnormalities (Table 2) and MS (Fig. 4) in patients with MAFLD. Meanwhile, individuals with more MS risk factors had a higher prevalence of MAFLD (Table 3), and patients with MS also had a higher prevalence of MAFLD than those without MS. These findings suggested that MAFLD is prone to coexist with systemic metabolic disorders, and a deep inner relationship between MAFLD and MS may exist in which the two diseases have a great influence and interaction on each other. Notably, we noticed that the prevalence of MS in females with MAFLD was significantly higher than that in males with MAFLD (Fig. 4), indicating that among patients with MAFLD, females may be more susceptible to MS than males, which warrants further investigation.

It was shown in our study that the prevalence of dyslipidaemia and hyperuricaemia was significantly higher in individuals with MAFLD than in those without MAFLD (Fig. 4). Dyslipidaemia is a well-known risk factor for NAFLD [3], and this can also be reflected in the sharp rise in the prevalence of MAFLD in perimenopausal and postmenopausal women in our study, which might be related to dyslipidaemia due to oestrogen deficiency (Fig. 2), indicating that dyslipidaemia may also be a risk factor for MAFLD. In the binary logistic regression analysis, uric acid was shown to be significantly correlated with MAFLD. Previous cross-sectional and prospective studies have found that elevated serum uric acid could independently predict an increased risk of NAFLD, even serum uric acid levels within the normal range were closely correlated with the presence of NAFLD independently [24–26]. Hence, combining the findings in our study with previous studies, serum uric acid might be considered an independent risk factor for MAFLD.

The present study revealed that individuals with MAFLD are more likely to have elevated liver enzymes, particularly elevated ALT, than those without MAFLD (Table 2), which indicates a higher proportion of abnormal liver function in individuals with MAFLD. Moreover, Table 4 shows that ALT was significantly correlated with MAFLD, and previous studies have shown that elevated ALT is associated with the progression of NAFLD into steatohepatitis and even liver fibrosis [27], indicating that elevated ALT might also have important clinical significance for MAFLD. Platelets are elevated during inflammation, and previous studies have found a linear correlation between platelet count and the severity of liver fibrosis in individuals with NAFLD [28]. In our study, we also found that platelet count was significantly correlated with MAFLD (Table 4), indicating that platelet count and ALT levels may be used as a reference indicator of MAFLD development and the resulting liver fibrosis. As some blood biomarkers, such as the NAFLD fibrosis score (NFS), have been used to assess the degree of liver fibrosis in NAFLD patients [29], more studies are also needed to build mathematical models on fibrosis biomarkers and explore a noninvasive fibrosis scoring system for MAFLD patients.

Although the occurrence of NAFLD is closely correlated with obesity, nonobese individuals may also suffer from NAFLD, particularly in the Asia–Pacific region [30]. In our study, the proportions of abnormal metabolic features in nonobese individuals with MAFLD were all significantly higher than those in nonobese individuals without MAFLD (Table 2), suggesting that metabolic disorders also play an important role in the occurrence of MAFLD in nonobese individuals. Meanwhile, between obese and nonobese MAFLD patients, the proportions of patients with elevated blood pressure and elevated liver enzymes were significantly higher in obese MAFLD patients, while the relationship between obesity and elevated liver enzymes, potential liver function impairment, and elevated blood pressure has also been described in previous articles [31, 32], suggesting that obese patients may have an increased risk of cardiovascular events and liver function impairment; however, no significant difference was found in the proportions of patients with abnormal blood lipids and elevated fasting glucose between the obese and nonobese MAFLD groups. These results indicate that even in nonobese MAFLD patients, there were already metabolic abnormalities in blood lipids and blood glucose levels that were comparable to those in obese MAFLD patients, which needs to be given sufficient attention.

Our study also has certain limitations. First, being a cross-sectional study, the natural course of MAFLD and causal relationships cannot be determined. Our study included a large sample size with a wide range of clinical data, making it possible to adjust for underlying confounding factors. Second, the diagnosis of MAFLD was based on ultrasonography, which might be partially insensitive to mild hepatic steatosis; however, ultrasonography has been widely used in epidemiological investigations of fatty liver because it is safe, noninvasive, and widely available; has acceptable sensitivity and specificity in the detection of hepatic steatosis [33]; and is also recommended as the first-line imaging method by the Association for the Study of the Liver (APASL) in the clinical guidelines for MAFLD [34]. Using ultrasound to screen for MAFLD might underestimate the prevalence of MAFLD; however, the possible underestimated value in our study has already shown the heavy burden of MAFLD in China, indicating that MAFLD in China should be given more attention. Third, certain selection bias may exist because the population who participated in the health examinations included in our study tended to be more concerned about their health. Fourth, some information was not available from the current health examination data, such as detailed medication history of participants and data on hepatitis virus and alcohol consumption. Further studies with subgroup analyses on viral hepatitis and alcohol consumption are needed. Last, because menopausal history was difficult to obtain in the population undergoing health examination, we artificially categorized menopausal status by age based on relevant research and the mean menopausal age of women, which might harbour information bias due to misclassification.

## Conclusions

Our study revealed a high prevalence of MAFLD within an urban Chinese population. The prevalence of MAFLD varies considerably between different groups based on sex, age, BMI and female menopausal status. An increased prevalence was found to be associated with obesity and multiple metabolic disorders, and individuals with MAFLD had a high prevalence of MS, dyslipidaemia, and hyperuricaemia. MAFLD tends to coexist with systemic metabolic disorders, the presence of MAFLD and MS interact with each other, and they may have a deep influence on each other. Moreover, nonobese individuals also suffer from MAFLD, which was also found to be closely correlated with metabolic disorders. We also confirmed the high proportion of elevated ALT in individuals with MAFLD. Multiple metabolic disorders, especially obesity, should be given more attention to prevent and better manage MAFLD. More research is needed to determine the potential mechanisms underlying the occurrence of MAFLD, and to better understand the relationship and causality between MAFLD and multiple metabolic disorders, which would provide crucial implications for the prevention and treatment of MAFLD.

## Data Availability

The datasets analysed during the current study are not publicly available because all data were recorded in the electronic medical record system of the Quality Control Center of Health Examination in Chongqing. However, they are available from the corresponding author on reasonable request.

## Abbreviations

MAFLD: Metabolic associated fatty liver disease
NAFLD: Nonalcoholic fatty liver disease
MS: Metabolic syndrome
BMI: Body mass index
HDL-C: High-density lipoprotein cholesterol
LDL-C: Low-density lipoprotein cholesterol
ALT: Alanine aminotransferase
AST: Aspartate aminotransferase
BUN: Blood urea nitrogen
WBC: White blood cell count
RBC: Red blood cell count
HCT: Hematokrit
OR: Odds ratio
CI: Confidence interval
NFS: NAFLD fibrosis score
